# Influence of Retainer Design on Shear Bond Strength in Fixed Partial Dentures: An In Vitro Comparative Study

**DOI:** 10.7759/cureus.112301

**Published:** 2026-07-08

**Authors:** Harshal R Gupta, Tony Thomas C, Manju V, Arimboor Maymol Francis, Sreeprabha G Mohan

**Affiliations:** 1 Prosthodontics, Amrita School of Dentistry, Amrita Vishwa Vidyapeetham, Kochi, IND

**Keywords:** fixed partial denture, modified overlay retainer, monolithic zirconia, resin-bonded fixed dental prosthesis, shear bond strength

## Abstract

Background: Resin-bonded fixed dental prostheses (RBFDPs) are considered conservative alternatives to conventional fixed partial dentures for replacing missing teeth. However, concerns regarding retention and debonding remain, particularly in posterior regions. This study evaluated the influence of different retainer designs on the shear bond strength (SBS) of fixed partial dentures.

Methods: Fifteen monolithic zirconia fixed partial dentures were divided into three groups: conventional overlay-retained FPD (OVL), modified overlay-retained FPD (MOVL), and conventional full-coverage crown-retained FPD (FCC) (n = 5 each). Standardized preparations were performed on epoxy resin dies, and all restorations were cemented using dual-cure resin cement. Shear bond strength testing was carried out using a universal testing machine, followed by failure mode analysis under a stereomicroscope. Statistical analysis was performed using the Kruskal-Wallis and Chi-square tests.

Results: Group C (FCC) showed the highest mean shear bond strength (1492.70 ± 87.25 N), followed by Group B (MOVL) (1201.60 ± 186.22 N), while Group A (OVL) demonstrated the lowest values (469.40 ± 190.57 N). A statistically significant difference was observed among the groups (p < 0.001). Adhesive failure predominated in Group A, whereas mixed failures were more common in Groups B and C. Failure mode distribution showed no statistically significant difference (p = 0.086).

Conclusion: The modified overlay-retained FPD demonstrated significantly higher shear bond strength than the conventional overlay design and showed performance comparable to the conventional full-coverage crown-retained prosthesis. Within the limitations of this in vitro study, the modified overlay design may serve as a conservative and clinically acceptable alternative for posterior fixed partial dentures.

## Introduction

Preservation of natural teeth should be the primary objective of every dentist. Just as the loss of even a small part of a finger or toe is considered significant, the loss of any portion of a tooth must be regarded as a serious injury requiring careful restoration. Tooth loss, whether due to recurrent caries, trauma, endodontic complications, or periodontal disease, can lead to functional impairment of the masticatory system [1]. Congenital absence of teeth, also known as dental agenesis, is among the most common dental anomalies. It can negatively affect both aesthetics and function, influencing self-esteem, communication, professional performance, and overall quality of life [2]. Loss of posterior teeth may further result in drifting of adjacent teeth, supra-eruption of opposing teeth, occlusal imbalance, and compromised masticatory efficiency. Therefore, timely replacement of missing teeth is essential not only to restore aesthetics and function but also to maintain overall oral health and occlusal stability.

Various treatment options are available for replacing a single missing tooth. Conventional metal-ceramic fixed partial dentures (FPDs) are widely used but have aesthetic limitations due to visible metal margins. All-ceramic FPDs offer improved aesthetics; however, their reduced strength and the need for aggressive tooth preparation restrict their use. They may also increase the risk of pulpal damage, root fractures, and eventual tooth loss. Implant-supported restorations provide a more conservative alternative, but their use may be limited by cost, surgical concerns, or medical contraindications [3]. In addition, certain clinical situations such as insufficient bone quantity, young age, systemic health conditions, or patient reluctance toward surgical procedures may further limit the applicability of implant therapy. Consequently, there remains a continued need for minimally invasive fixed prosthodontic treatment options that preserve sound tooth structure while providing adequate function and aesthetics.

Resin-bonded fixed dental prostheses (RBFDPs) were first introduced by Rochette in the 1970s as a conservative and temporary option for replacing missing anterior teeth. In the 1980s, the Maryland bridge was developed using electrolytic etching of nickel-chromium alloys, which improved micromechanical bonding between resin cement and the metal surface [4,5]. The main advantage of RBFDPs is their minimally invasive nature, as they require little or no tooth preparation compared with conventional fixed dental prostheses. Resin-bonded fixed dental prostheses (RBFDPs) have demonstrated high success rates, particularly in anterior regions. They are conservative, preserve tooth structure, and are suitable for younger patients [6]. Furthermore, the reduced preparation design minimizes postoperative sensitivity and preserves enamel, thereby improving adhesive bonding and long-term retention.

With advancements in adhesive technology and increasing aesthetic demands, tooth-colored partial coverage restorations such as inlays, onlays, and overlays have gained prominence [7,8]. Improvements in computer-aided design/computer-aided manufacturing (CAD/CAM) technology and high-strength ceramic materials have contributed to enhanced precision, marginal adaptation, and fracture resistance of contemporary restorations. These restorations allow preservation of tooth structure while reinforcing weakened teeth [9]. Overlay restorations, in particular, provide cuspal coverage while maintaining a more conservative preparation design compared with full-coverage crowns. Such restorations may distribute occlusal stresses more favorably and reduce the amount of unnecessary removal of healthy dental tissue.

Although full-coverage crowns remain the gold standard for fixed partial denture (FPD) retainers, they require extensive tooth reduction and may increase the risk of periodontal and pulpal complications [10]. To overcome these limitations, the present study introduces a modified overlay-retained fixed partial denture (MOVL) design intended to improve retention and resistance while preserving maximum tooth structure.

The objectives of this study are to compare the shear bond strength (SBS) of three retainer designs for posterior fixed partial dentures (conventional overlay-retained (OVL), modified overlay-retained (MOVL), and conventional full-coverage crown-retained (FCC) prostheses); to compare the failure modes of these three retainer designs following shear bond strength testing; and to determine whether the modified overlay-retained design provides improved bonding performance while maintaining a conservative tooth preparation compared with the conventional overlay design, and whether its performance approaches that of the conventional full-coverage crown-retained prosthesis.

The primary outcome measure was shear bond strength, while failure mode analysis served as the secondary outcome measure. The findings are intended to provide evidence regarding the clinical potential of the modified overlay-retained design for posterior resin-bonded fixed partial dentures.

## Materials and methods

Sample size calculation and sampling technique

**Figure 1:**
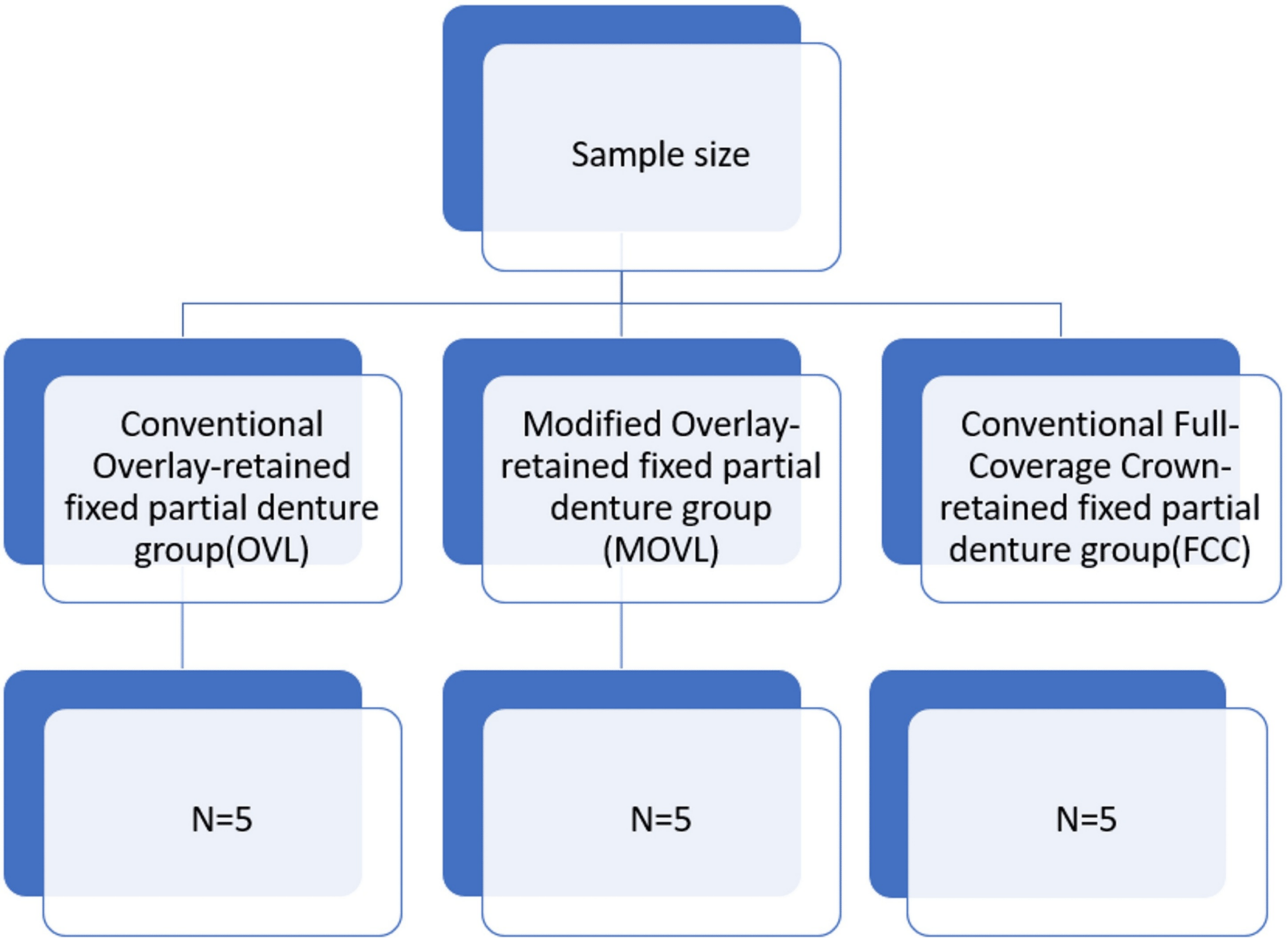
Flowchart illustrating the distribution of the total study sample into the three experimental groups used in the present in vitro comparative study The overall sample size was equally allocated into three groups, with each group consisting of five specimens (N = 5). Group A comprised the OVL group, Group B included the MOVL group, and Group C consisted of the FCC group. OVL: conventional overlay-retained fixed partial denture, MOVL: modified overlay-retained fixed partial denture, FCC: conventional full-coverage crown-retained fixed partial denture Picture credits: Dr. Harshal R. Gupta The flowchart was created using Microsoft PowerPoint (Microsoft Corporation, Redmond, WA).

The sample size for the present in vitro comparative study was estimated based on previously published shear bond strength data on resin-bonded fixed dental prostheses and retainer designs [11]. The sample size was calculated using the formula for comparison of means in one-way analysis of variance (ANOVA) studies, where n represents the minimum sample size per group, σ the estimated standard deviation, d the expected mean difference, Zα/2 the standard normal variate at 95% confidence interval (1.96), and Zβ the standard normal variate at 80% power (0.84). Based on the effect size obtained from Abdussalam et al. [11], five specimens per group were considered sufficient, resulting in a total sample size of 15 (Figure 1). A purposive non-probability sampling technique was used, and equal allocation of specimens was performed among the three study groups.

Inclusion and exclusion criteria

The inclusion criteria consisted of standardized mandibular typodont models with a missing mandibular first molar, uniformly prepared abutment teeth, monolithic zirconia restorations fabricated using identical CAD/CAM protocols, defect-free epoxy resin dies, and specimens cemented using a standardized adhesive protocol. Samples with surface defects, cracks, improper marginal fit, fabrication distortion, cementation errors, incomplete seating, or fracture before testing were excluded from the study.

Data selection and standardization process

To minimize confounding variables and ensure reproducibility, all specimens were standardized throughout the experimental procedure. A single mandibular typodont model was used for all preparations, and all tooth preparations were performed by a single operator using standardized burs and verified using a periodontal probe and putty index. All restorations were fabricated using identical zirconia materials, CAD/CAM software, and milling parameters. Surface treatment, cementation procedures, curing cycles, and shear bond testing conditions were standardized across all groups to reduce operator and instrument bias. Epoxy resin dies were selected because of their validated dentin-analog behavior and dimensional uniformity during mechanical testing [12-14].

Outcome measures and evaluation criteria

The primary outcome measure evaluated in the present study was shear bond strength (SBS), which was recorded as a continuous quantitative variable and expressed in Newtons (N) using a universal testing machine. Failure mode analysis was assessed using a categorical failure classification scale under stereomicroscopic evaluation at 10× magnification, according to the criteria described by Holmer et al. [15]. Based on the extent of interfacial bond disruption, failures were classified as adhesive failure (>75% bond loss at the interface), mixed failure (combined adhesive and cohesive failure), and cohesive/substrate failure (<25% interfacial bond loss with predominant substrate fracture). These evaluation criteria have been widely used in adhesive dentistry studies assessing zirconia-resin bonding systems and debonding behavior [15].

Existing literature indicates a lack of comparative data on the shear bond strength and failure modes of conventional fixed partial dentures (FPDs) versus modified overlay-retained FPDs in the posterior region. To address this gap, a total of 15 samples were included in the study, with 5 samples assigned to each group [11]. The study consisted of three groups: Group A, conventional overlay-retained FPD (OVL); Group B, modified overlay (overlay with proximal extension)-retained FPD (MOVL); and Group C, conventional full-coverage crown (FCC)-retained FPD. All prostheses were fabricated using monolithic zirconia as the material of choice. The restorations were cemented onto epoxy resin dies using resin cement under standardized conditions. The experimental procedures were conducted at Praj Dental Laboratory, Pune, Maharashtra, India, over a period of one month (April 2026). All samples were subjected to shear bond strength testing using a universal testing machine. Following debonding, failure mode analysis was performed to evaluate the nature of bond failure.

A mandibular jaw model with ivorine teeth (Nissin) was used to simulate a clinical scenario involving a missing first molar, with the second premolar and second molar serving as abutments. The first molar was removed, and the space was filled with modeling wax (MAARC Modelling Wax, MAARC Dental, India) to maintain the edentulous space during sample preparation.

For Group A (OVL), a 2 mm occlusal reduction was performed, and finish lines were prepared at the same depth. Initial preparation was done using a round bur, followed by finishing burs to refine the margins. The preparation was assessed using a periodontal probe and putty index to ensure accuracy.

For Group B (MOVL), a 2 mm occlusal reduction was carried out along with axial reduction. Additionally, a 2 mm mesial axial reduction was done on the second molar and a distal axial reduction on the second premolar. Shoulder margins, similar to those in the other groups, were prepared.

For Group C (FCC), tooth preparation involved a 2 mm occlusal reduction, followed by axial, buccal (labial), and lingual reductions. A shoulder finish line was established. The depth of preparation was verified using a periodontal probe and a putty index (Hydrorise, Zhermack, Badia Polesine, Italy) fabricated prior to tooth preparation.

**Figure 2:**
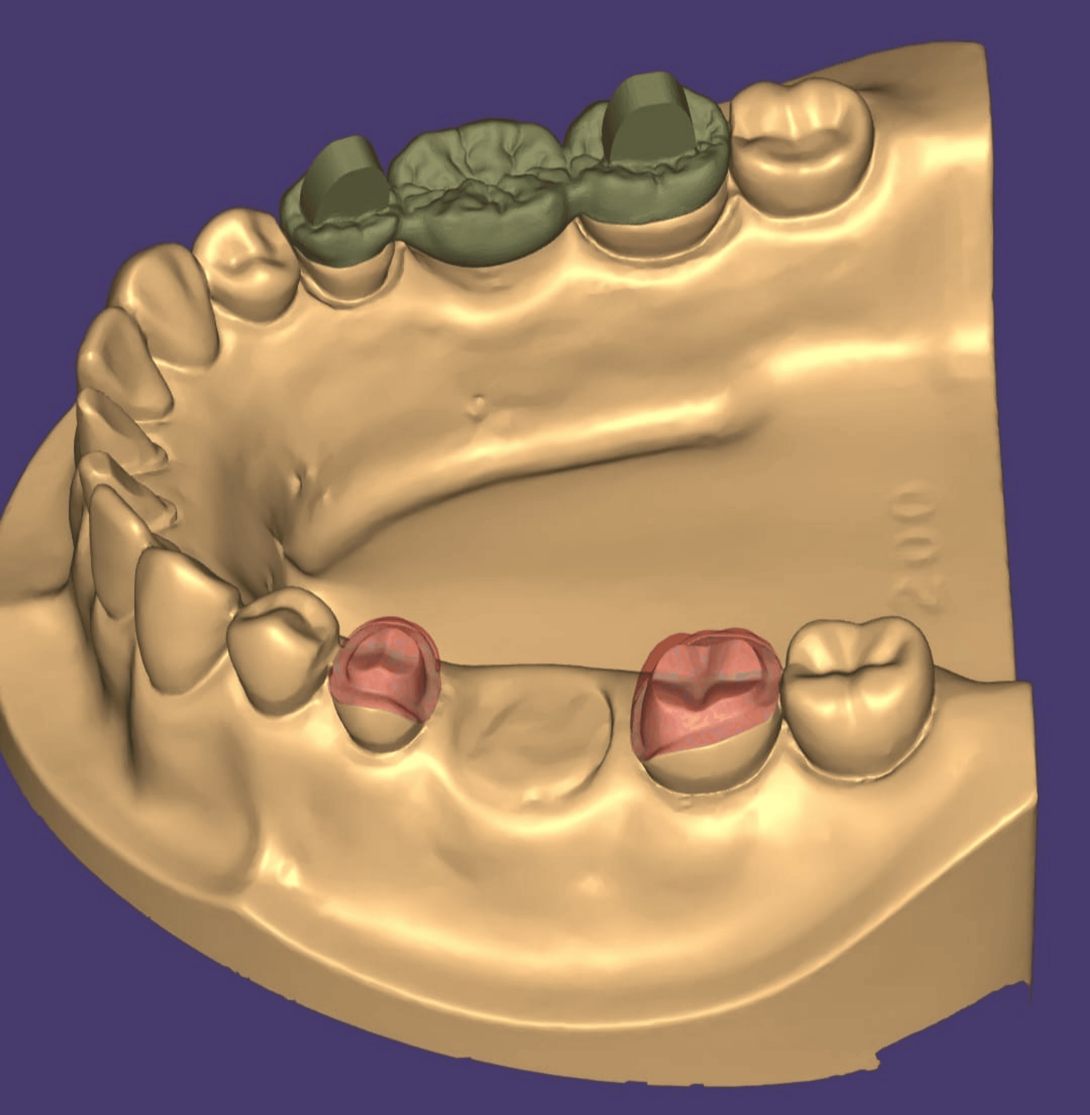
Digital design of the MOVL MOVL: modified overlay-retained fixed partial denture Digital designing of this restoration was performed using exocad DentalCAD software developed by exocad GmbH, Darmstadt, Germany. Picture credits: Dr. Harshal R. Gupta

**Figure 3:**
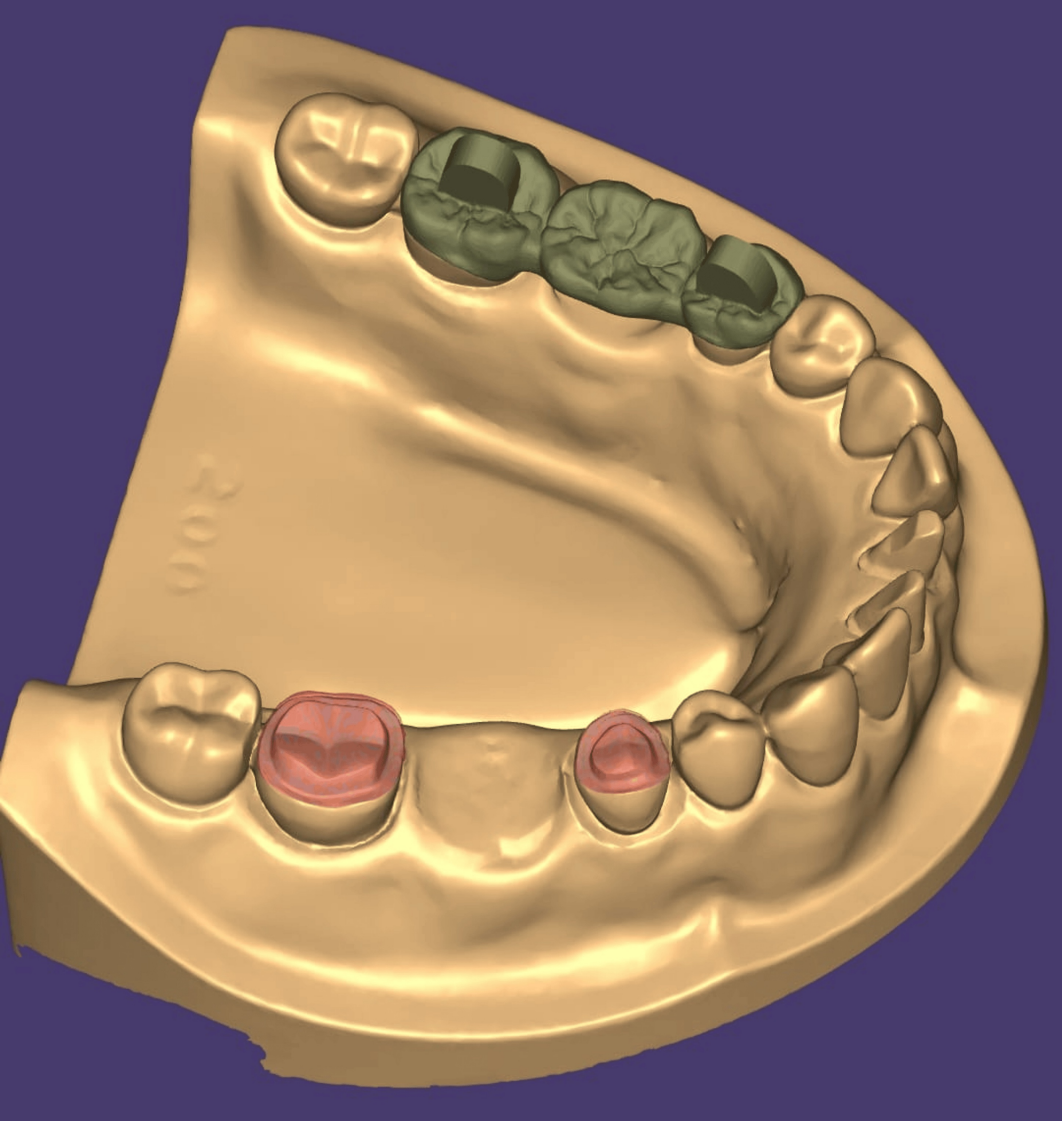
Digital design of the OVL OVL: conventional overlay-retained fixed partial denture Digital designing of this restoration was performed using exocad DentalCAD software developed by exocad GmbH, Darmstadt, Germany. Picture credits: Dr. Harshal R. Gupta

All tooth preparations were performed using diamond burs (Shofu, Kyoto, Japan). The prepared mandibular model was then scanned using a laboratory scanner (Medit, Seoul, South Korea), and stereolithography (STL) files were generated for each group. The preparations were evaluated for the presence of undercuts prior to prosthesis design. The prostheses were digitally designed using DentalCAD 3.0 Galway software (Exocad GmbH, Darmstadt, Germany) (Figure 2 and Figure 3). Fabrication was carried out using a five-axis milling machine (IMES-ICORE 350i, imes-icore GmbH, Eiterfeld, Germany) with monolithic zirconia blanks (Apsara Zirconia, India). The milled restorations were inspected for fit, surface irregularities, and cracks.

Fabrication of Epoxy Resin Dies

Epoxy resin dies were fabricated for this study, as they are widely accepted as a dentin-analog material in experimental research. Their use allows standardization of sample dimensions and geometry by eliminating the variability associated with natural tooth anatomy, thereby simplifying the methodology and avoiding the requirement for ethical approval. Previous research demonstrated that crowns luted onto epoxy resin and natural teeth exhibit comparable mechanical behavior, including similar tensile stress distribution and peak stress values, supporting their validity as a substitute material [12-14].

For the fabrication of epoxy dies, multiple putty indices were prepared for each group and subsequently boxed using modeling wax. Epoxy resin (EPOKE Art Epoxy Resin, EPOKE Art, India) and hardener, supplied as a two-component system, were mixed according to the manufacturer’s instructions in a 2:1 volume ratio. The mixture was briefly flamed to eliminate entrapped air bubbles and then allowed to polymerize at room temperature for 48 hours. After complete curing, the dies were retrieved and carefully inspected for any surface defects such as voids or nodular irregularities before further use.

Bonding and Cementation

Prior to cementation, the epoxy resin dies were surface-treated by airborne particle abrasion using 50 µm aluminium oxide for 10 seconds at a pressure of 2.5 bar. The treated surfaces were then cleaned with 96% alcohol to remove any residual contaminants [9]. To enhance bonding, a zirconia primer (Z-Prime Plus, BISCO Inc., Schaumburg, IL) was applied to the intaglio surfaces of all zirconia prostheses. This primer contains a combination of functional monomers, namely, 10-methacryloyloxydecyl dihydrogen phosphate (MDP) phosphate monomer and biphenyl dimethacrylate (BPDM) carboxylate monomer, which act synergistically to improve chemical adhesion and increase overall bond strength.

Cementation was carried out using a dual-cure adhesive resin cement (3M™ ESPE™ RelyX™ U200 Automix, 3M, St. Paul, MN). The two components were dispensed and mixed according to the manufacturer’s instructions. The mixed cement was applied to the intaglio surface of the monolithic zirconia restorations, which were then seated onto the prepared epoxy dies under standardized conditions. An initial light curing (tack cure) was performed for 3 seconds using an LED curing unit (Woodpecker, Guilin Woodpecker Medical Instrument Co., Ltd., Guilin, China) to facilitate removal of excess cement. After thorough cleanup, final light polymerization was carried out for 40 seconds to ensure complete curing. This procedure was consistently followed for all 15 samples, comprising three groups with five specimens in each group.

Testing Shear Bond Strength Test (SBS Test)

Shear bond strength testing was performed for all 15 specimens using a universal testing machine (UNITEST 10, Instron Corporation, Norwood, MA). The machine was operated at a crosshead speed of 1 mm/min under ambient room temperature conditions. Each specimen was secured in a custom-made jig to ensure proper alignment. A chisel-shaped loading applicator was positioned to deliver force at the bonding interface, applying a shear load centrally until debonding of the retainer occurred [11]. The testing machine was calibrated in accordance with International Organization for Standardization (ISO) standards. Load cell accuracy (±1%) was verified using standard weights, and the crosshead speed was validated using a digital tachometer. During testing, load-deflection curves and the maximum load at failure were automatically recorded using the system software (Bluehill Lite, Instron, Canton, MA). The force at failure was measured in Newtons (N).

Failure Mode Analysis

Following shear bond testing, all debonded specimens were evaluated to determine the mode of failure. The fractured surfaces were examined under a stereomicroscope (Model XTL-3400E, Ceti Scientific Instruments, Belgium) at 10× magnification.

Based on the extent and nature of bond disruption, the failures were categorized into three types: adhesive failure, more than 75% of the surface exhibited loss of bond at the interface; mixed failure, approximately 50% of the surface showed a combination of bond failure and substrate fracture; and substrate failure, less than 25% of the surface demonstrated bond loss, indicating predominant failure within the material. Image analysis was further performed using MVIG 2005 image analysis software (PRAJ Dental Laboratory, Pune, India) to support the evaluation [15].

Fabrication of epoxy resin dies, tooth preparation procedures, digital designing and fabrication of monolithic zirconia crowns, and bonding and cementation procedures were performed at the Department of Prosthodontics, Amrita School of Dentistry, Amrita Vishwa Vidyapeetham, Kochi, India. The shear bond strength testing and failure mode analysis were subsequently carried out at Praj Dental Laboratory, Pune, Maharashtra, India, using a universal testing machine and stereomicroscopic evaluation under standardized laboratory conditions.

The study was conducted over a one-month period in April 2026. During the initial phase, digital scanning and CAD design of the retainer preparations and prostheses were completed. This was followed by fabrication and milling of monolithic zirconia restorations using a five-axis milling machine. Subsequently, epoxy resin dies were fabricated, and standardized tooth preparation, bonding, and cementation procedures were performed at the Department of Prosthodontics, Amrita School of Dentistry, Amrita Vishwa Vidyapeetham, Kochi, India. In the final phase of the study, shear bond strength testing and failure mode analysis were carried out at Praj Dental Laboratory, Pune, Maharashtra, India, using a universal testing machine and stereomicroscopic evaluation under standardized laboratory conditions.

## Results

**Figure 4:**
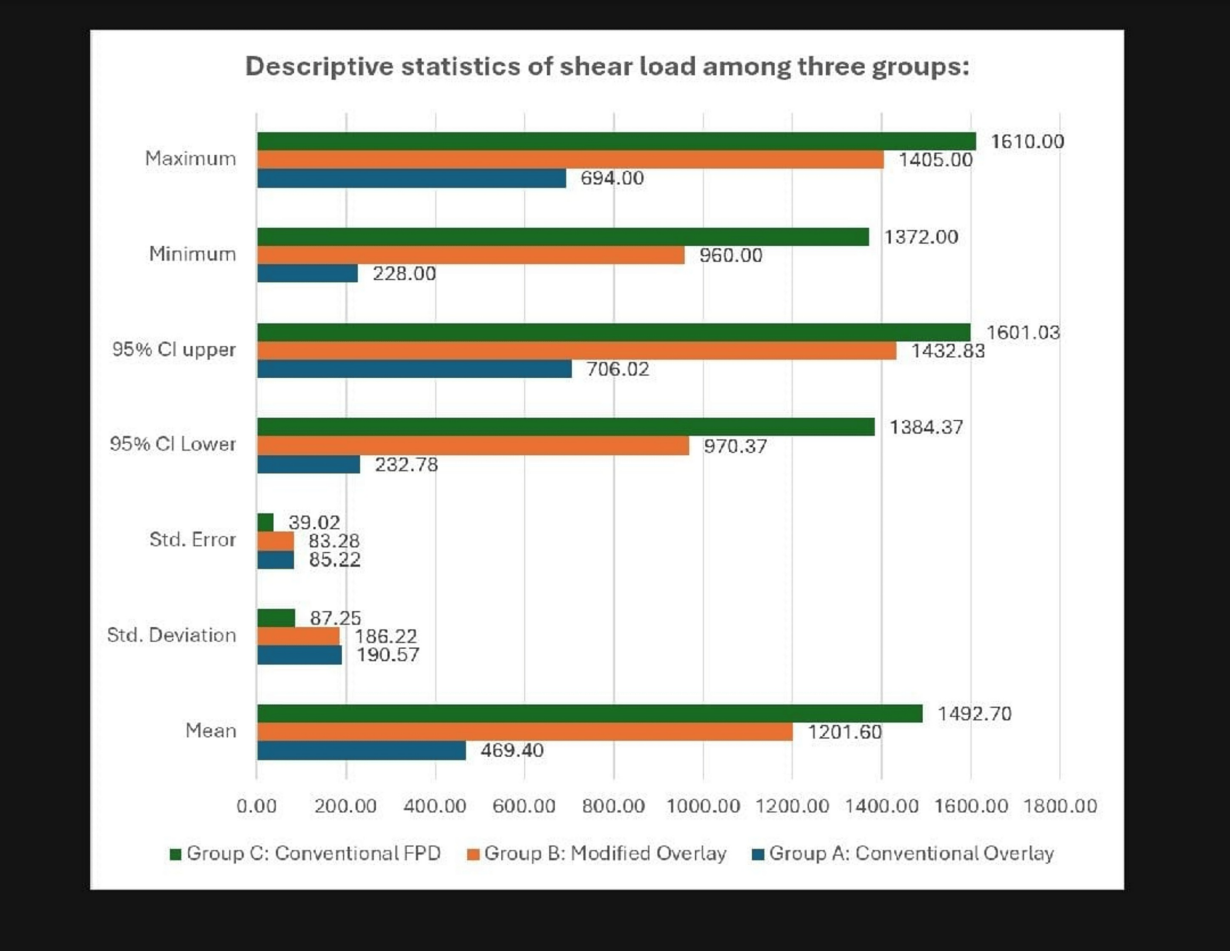
Descriptive statistics of shear load among the three groups A: conventional overlay, B: modified overlay, C: conventional FPD FPD: fixed partial denture The graph was created using IBM SPSS Statistics for Windows version 25.0 (IBM Corporation, Armonk, NY). Picture credits: Dr. Harshal R. Gupta

Table 1 presents the descriptive statistics of shear load among the three study groups. A progressive increase in shear load was observed from Group A to Group C. Group C (conventional FPD) demonstrated the highest mean shear load (1492.70 ± 87.25 N) with the least variability, followed by Group B (modified overlay) (1201.60 ± 186.22 N). Group A (conventional overlay) exhibited the lowest mean shear load (469.40 ± 190.57 N) and the greatest variability in measurements. It is graphically represented in Figure 4.

**Table 1:** Descriptive statistics of shear load among the three study groups Group A: conventional overlay, Group B: modified overlay, Group C: conventional full-coverage crown-retained FPD Values expressed as mean ± SD. Statistical analysis performed using the Kruskal-Wallis test followed by pairwise intergroup comparison. n = 5 specimens per group (33.3% of total study sample per group). SD: standard deviation, FPD: fixed partial denture Table credits: Dr. Harshal R. Gupta

Group	Mean	SD	Standard error	95% confidence interval for mean		Minimum	Maximum
				Lower	Upper		
Group A	469.40	190.57	85.22	232.78	706.02	228.00	694.00
Group B	1201.60	186.22	83.28	970.37	1432.83	960.00	1405.00
Group C	1492.70	87.25	39.02	1384.37	1601.03	1372.00	1610.00

**Figure 5:**
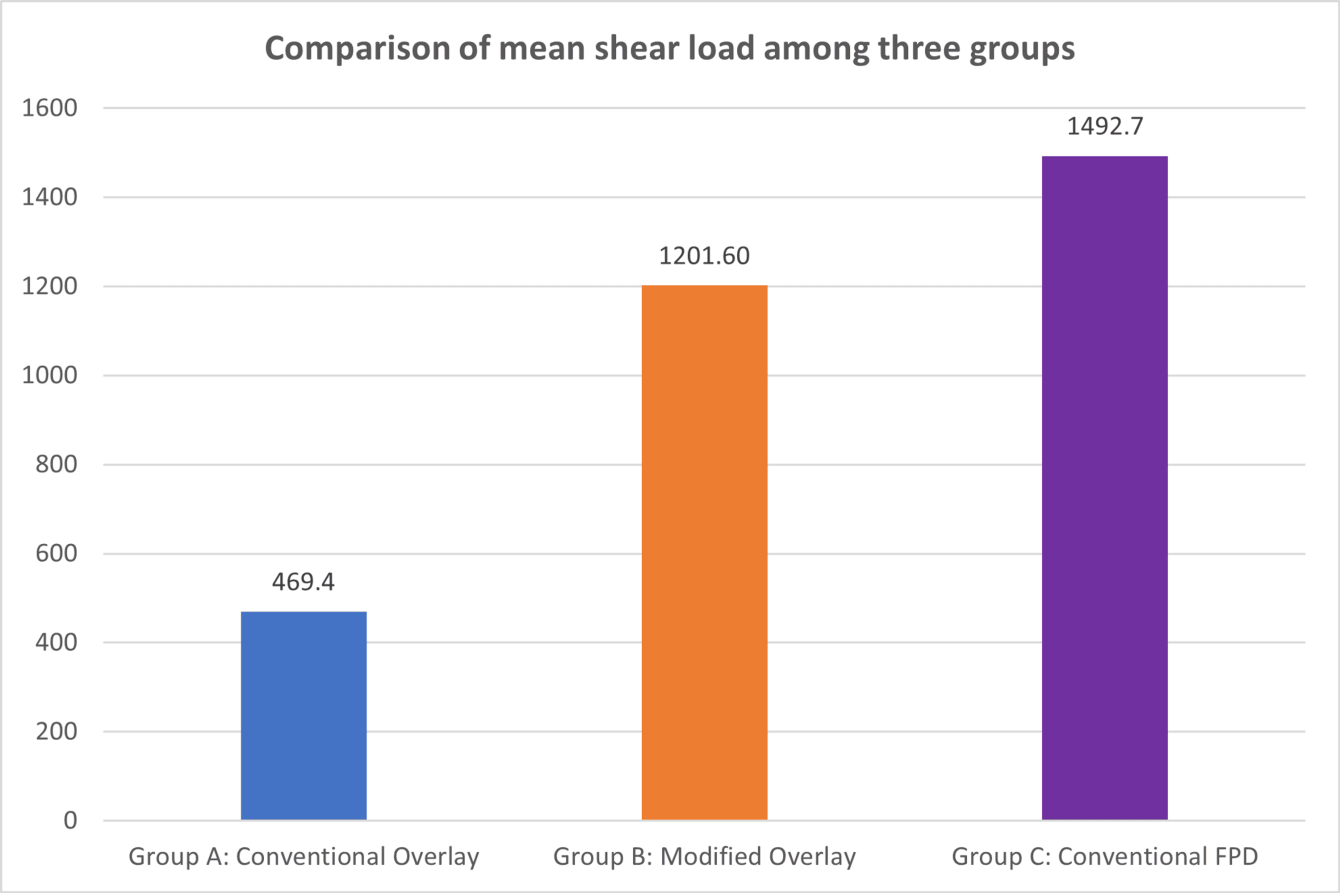
Comparison of mean shear load among the three groups A: conventional overlay, B: modified overlay, C: conventional FPD FPD: fixed partial denture Statistical analysis was performed using the Kruskal-Wallis test. The graph was created using IBM SPSS Statistics for Windows version 25.0 (IBM Corporation, Armonk, NY). Picture credits: Dr. Harshal R. Gupta

Figure 5 shows the comparison of mean shear load values among the groups. Statistical analysis using the Kruskal-Wallis test revealed a highly significant difference among the three groups (H = 53.049, p < 0.001), indicating that the type of restoration significantly influenced shear load resistance. Intergroup comparison demonstrated highly significant differences between Group A and Group B (p < 0.001) and between Group A and Group C (p < 0.001). A statistically significant difference was also observed between Group B and Group C (p = 0.037). Overall, Group C exhibited the highest shear load resistance, followed by Group B, while Group A demonstrated the least resistance.

**Figure 6:**
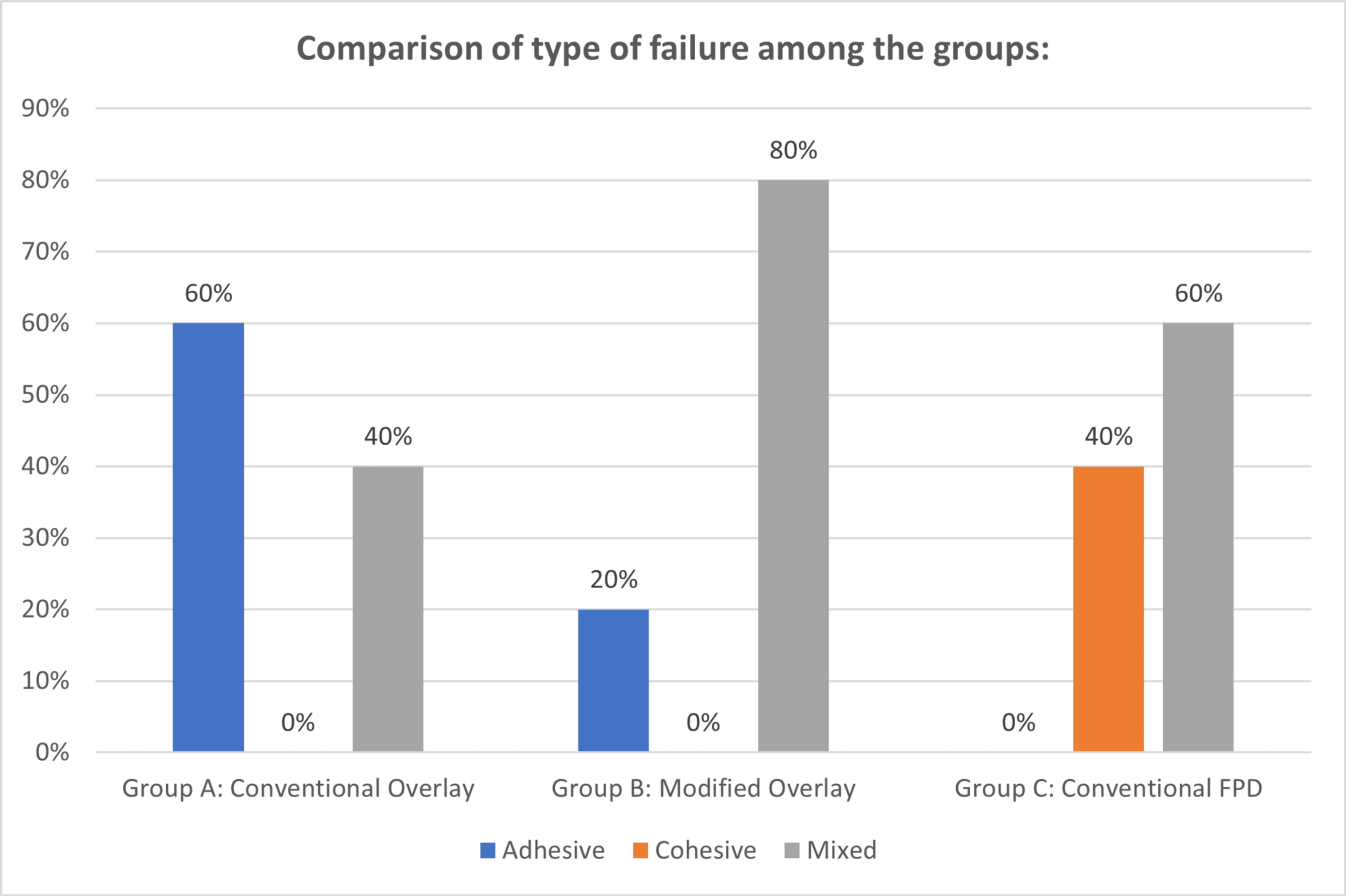
Comparison of failure among the three groups A: conventional overlay, B: modified overlay, C: conventional FPD FPD: fixed partial denture The graph was created using IBM SPSS Statistics for Windows version 25.0 (IBM Corporation, Armonk, NY). Picture credits: Dr. Harshal R. Gupta

Table 2 illustrates the distribution of failure types among the study groups. Adhesive failure was predominant in Group A (60%), whereas mixed failure was the most common mode in Group B (80%) and Group C (60%). Cohesive failure was observed only in Group C (40%). However, Chi-square analysis revealed no statistically significant difference in the distribution of failure patterns among the groups (χ² = 8.167, p = 0.086), indicating that the variation in failure modes was not statistically significant. This is graphically represented in Figure 6.

**Table 2:** Description of failure types among the groups A: conventional overlay, B: modified overlay, C: conventional FPD FPD: fixed partial denture Distribution of failure modes among the three experimental groups. Data presented as frequency (number) and percentage (%). Statistical analysis performed using the Chi-square test. n = 5 specimens per group (33.3% of total study sample per group). Table credits: Dr. Harshal R. Gupta

Failure	Group A		Group B		Group C	
	Number	%	Number	%	Number	%
Adhesive	3	60%	1	20%	0	0%
Cohesive	0	0%	0	0%	2	40%
Mixed	2	40%	4	80%	3	60%
Total	5	100%	5	100%	5	100%
Chi-square test, p-value	χ^2 ^= 8.167, p = 0.086 NS					

## Discussion

The retainer designs of RBFDP to replace a missing mandibular first molar were evaluated. The three groups included conventional overlay crown-retained RBFDP (OVL), modified overlay crown-retained RBFDP (MOVL), and full-coverage crown-retained RBFDP (FCC). Comparison among the groups using the Kruskal-Wallis test demonstrated a highly significant difference in shear load values (H = 53.049, p < 0.001). Intergroup analysis revealed highly significant differences between Group A and Group B (p < 0.001) and between Group A and Group C (p < 0.001). A statistically significant difference was also observed between Group B and Group C (p = 0.037). Overall, the conventional full-coverage crown-retained FPD exhibited the highest shear load resistance, followed by the modified overlay design, whereas the conventional overlay design demonstrated the lowest resistance.

The selection of treatment modalities for replacement of missing teeth includes orthodontic therapy, implant-supported restorations, and prosthodontic rehabilitation. Among these options, preservation of natural tooth structure should remain the primary consideration during treatment planning. Contemporary restorative dentistry strongly emphasizes minimally invasive procedures through careful diagnosis, proper case selection, and conservative preparation designs aimed at improving the long-term prognosis of restorations. In this regard, all-ceramic resin-bonded fixed dental prostheses (RBFDPs) have emerged as a conservative and aesthetically favorable alternative for the rehabilitation of partial edentulism.

Compared with conventional fixed dental prostheses, RBFDPs are associated with fewer biologic complications because they require minimal tooth preparation. Failures associated with conventional FPDs often result in irreversible consequences such as secondary caries, pulpal damage, apical pathology, periodontal complications, and eventual loss of abutment teeth due to extensive preparation and subgingival margin placement [16]. In contrast, failures associated with resin-bonded prostheses are generally less destructive and more manageable, with debonding being the most common complication rather than catastrophic fracture or irreversible biological damage. In younger patients, preservation of enamel and maintenance of pulpal vitality are particularly important, making minimally invasive retainers highly advantageous [17]. Preservation of enamel also contributes to improved adhesive bonding because enamel bonding is considered more predictable and durable than dentin bonding, thereby improving retention and long-term prosthesis survival.

To enhance the retention and longevity of RBFDPs, various tooth preparation modifications and retentive features have been proposed. Additional preparation features such as grooves, boxes, lingual rests, and proximal extensions have been recommended to improve resistance form and seating accuracy [18]. However, incorporation of these features may increase the amount of healthy tooth reduction, thereby conflicting with the principles of conservative dentistry [19]. Consequently, there has been continued interest in developing retainer designs that balance adequate mechanical retention with maximal preservation of tooth structure.

The inlay-retained fixed dental prosthesis (IRFDP) was introduced as a conservative alternative to full-coverage retainers. Clinical studies have demonstrated favorable survival rates for IRFDPs, with reported success rates of approximately 92.6% at 3 years and 87.9% at 5 years [20]. Despite these encouraging outcomes, both biological and technical complications have been documented [21,22]. Biological complications commonly include postoperative sensitivity and secondary caries formation due to preparation extension into dentin, whereas technical complications primarily involve debonding of retainers, connector fracture, and ceramic chipping. These complications highlight the importance of optimizing retainer design and connector dimensions to improve stress distribution and enhance prosthesis longevity.

Considering these limitations, the present study introduced a novel modified overlay-retained resin-bonded fixed dental prosthesis (MOVL). The rationale behind the MOVL design was to preserve maximum tooth structure while simultaneously providing adequate retention, resistance form, and connector thickness. In this preparation design, reduction is limited mainly to the occlusal and proximal axial surfaces, thereby conserving a substantial amount of healthy enamel and dentin. Preservation of enamel may improve bonding durability and reduce the risk of postoperative sensitivity and pulpal complications. At the same time, the preparation design permits adequate connector dimensions, which are critical for improving fracture resistance and reducing stress concentration within the prosthesis.

Another important advantage of the MOVL design is its flexibility in long-term treatment planning. In situations where repeated debonding or prosthesis failure occurs, the conservative preparation can subsequently be modified and converted into a conventional full-coverage crown-retained prosthesis without excessive additional tooth reduction. This staged treatment approach allows clinicians to initially adopt conservative restorative principles while still preserving future treatment options if required.

The findings of the present study demonstrated that the MOVL group exhibited significantly higher shear bond strength values than the conventional overlay-retained design and values approaching those of the conventional full-coverage crown-retained prosthesis. The improved performance observed in the MOVL group may be attributed to increased bonding surface area, improved stress distribution, and enhanced resistance form provided by the proximal extension design. The conventional overlay design demonstrated the lowest shear resistance, possibly because of limited retentive features and reduced bonding surface area, which may increase susceptibility to debonding under functional loading.

The conventional full-coverage crown-retained prosthesis exhibited the highest overall shear load resistance among all groups. This finding may be explained by the increased axial wall height, circumferential coverage, and larger surface area available for cementation, which collectively improve retention and mechanical stability. However, despite superior mechanical performance, full-coverage retainers require extensive tooth preparation and irreversible removal of healthy tooth structure, which may compromise pulpal health and periodontal integrity over time.

The modified overlay design demonstrated mechanical behavior comparable to the conventional full-coverage crown-retained design while maintaining a substantially more conservative preparation approach. This finding is clinically relevant because it suggests that conservative retainer designs may provide sufficient resistance to debonding and fracture while preserving healthy tooth structure. Such conservative approaches may be especially beneficial in younger individuals and patients with intact abutment teeth, where preservation of enamel and pulpal vitality is of paramount importance.

The failure mode analysis performed in the present study further supports the mechanical performance of the modified overlay design. The predominance of mixed and cohesive failures in the MOVL and FCC groups may indicate stronger adhesive bonding and improved stress distribution within the prosthesis complex. In contrast, the conventional overlay group demonstrated a greater tendency toward adhesive failures, suggesting weaker interfacial bonding and lower resistance to shear forces. These observations reinforce the importance of preparation design in determining both retention and biomechanical behavior of resin-bonded prostheses.

The improved bonding performance observed in the present study may also be associated with the use of monolithic zirconia restorations and standardized adhesive cementation protocols. Advances in zirconia surface treatment and adhesive technology have significantly enhanced the bonding reliability and fracture resistance of all-ceramic restorations. In addition, CAD/CAM fabrication enables improved marginal adaptation, standardized restoration thickness, and precise connector dimensions, which collectively contribute to improved mechanical behavior and prosthesis longevity.

Limitations

Despite the promising results observed in this investigation, the findings should be interpreted within the limitations of an in vitro study design. The oral environment is highly complex and dynamic, involving temperature fluctuations, humidity, salivary enzymes, pH changes, and multidirectional occlusal loading patterns that cannot be completely replicated under laboratory conditions [16]. Furthermore, cyclic fatigue loading and thermocycling were not performed in the present study, which limits assessment of long-term clinical durability. The relatively small sample size may also reduce the generalizability of the findings.

The use of epoxy resin dies, although widely accepted as dentin analogs in mechanical testing studies, may not completely reproduce the biomechanical behavior of natural teeth and periodontal ligament support [12-14]. Additionally, only one zirconia material and one adhesive cementation protocol were evaluated. Therefore, the results may not be directly extrapolated to other ceramic systems, restorative materials, or adhesive techniques. Future investigations incorporating larger sample sizes, thermomechanical aging, cyclic loading, finite element analysis, and clinical follow-up studies are necessary to validate the long-term clinical applicability of modified overlay-retained fixed dental prostheses [20,22].

Future perspectives

Future research should also focus on evaluating different restorative materials such as lithium disilicate ceramics, translucent zirconia, and hybrid ceramics in conjunction with conservative retainer designs. Investigation of alternative surface treatment protocols and adhesive systems may provide additional insights into improving bond durability and reducing technical complications such as debonding and connector fracture. Moreover, advancements in digital workflow technologies and computer-aided stress analysis may further optimize preparation geometry and biomechanical performance of minimally invasive posterior resin-bonded prostheses in clinical practice.

## Conclusions

A statistically significant difference was observed among the three retainer designs with respect to shear bond strength and failure mode analysis. Among the evaluated groups, the conventional full-coverage crown-retained fixed dental prosthesis (FCC) demonstrated the highest shear bond strength values, followed closely by the modified overlay (MOVL) design, while the conventional overlay design exhibited the lowest values. Although the FCC group showed slightly superior mechanical performance, the MOVL design demonstrated clinically acceptable bonding characteristics and mechanical stability, suggesting its potential as a conservative alternative for three-unit fixed partial dentures within the limitations of this in vitro study. The principal advantage of the MOVL design lies in its minimally invasive preparation approach, which preserves a substantial amount of healthy tooth structure while still maintaining adequate connector thickness necessary for fracture resistance and structural durability. Furthermore, in cases of repeated prosthesis failure or debonding, the MOVL preparation design allows subsequent conversion into a conventional full crown-retained fixed dental prosthesis with minimal additional tooth reduction, thereby providing flexibility for future restorative treatment planning while adhering to conservative restorative principles.
